# Concomitant use of dupilumab with glucocorticoid in bullous pemphigoid reduces disease severity: A preliminary study

**DOI:** 10.1002/iid3.924

**Published:** 2023-07-25

**Authors:** Lingyu Hu, Ruiting Huang, Fuqiong Jiang, Shuqiong You, Qian Wu

**Affiliations:** ^1^ Department of Dermatology The Second Affiliated Hospital of Kunming Medical University Kunming Yunnan China

**Keywords:** blood coagulation function, dupilumab biologics, eosinophils, IL‐13, IL‐4, immunoglobulin E, pemphigoid, treatment

## Abstract

**Objective:**

To retrospectively analyze the efficacy and safety of dupilumab in the treatment of bullous pemphigoid.

**Methods:**

From October 2020 to October 2022, the medical records of patients with bullous pemphigoid who were treated with dupilumab in our department were collected retrospectively to analyze the therapeutic effect and changes in laboratory indexes.

**Results:**

The records of a total of 11 patients with bullous pemphigoid who were treated with dupilumab was reviewed. Within 2 weeks of the treatment, 10 (90.9%) of the 11 patients had complete or substantial control of the disease. The BPDAI scores of the patients decreased from baseline 113 (62, 181) to 37 (6, 130) at 2 weeks (*p* = .001) and 4 (0, 37) at 12 weeks after treatment (*p* < .001). In the 11 patients treated with dupilumab, the relief time of pruritus was 0–3 days (0.5, 7) days, and the pruritus was significantly alleviated after 2 weeks (*t* = 15.925, *p* < .001). The DLQI score decreased from (25.5 ± 2.5) before treatment, to (11.8 ± 4.4) at 2 weeks (*t* = 10.764, *p* < .001) and (2.1 ± 1.9) at 12 weeks (*t* = 30.038, *p* < .001). The patients had high eosinophil counts, high serum IgE levels, low serum total protein levels, and abnormal blood coagulation function. The aforementioned indicators gradually returned to normal after treatment. No adverse reactions occurred during the treatment.

**Conclusion:**

Dupilumab can effectively control the condition of bullous pemphigoid, efficiently relieve pruritus symptoms, and is relatively safe.

## Graphical abstract

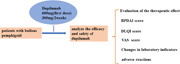

## 1 BACKGROUND

Bullous pemphigoid (BP) is an autoimmune subepidermal vesicular disease wherein the autoantibodies destroy the connection between the epidermis and basement membrane, causing blisters and bullae. The incidence of BP is mainly among the elderly. The number of patients is increasing as society ages. Its typical rash is erythema or tension bullae on otherwise normal‐looking skin, and patients frequently experience severe pruritus. Anti‐inflammatory drugs, glucocorticoids, immunosuppressants, and potent topical hormones are currently the main treatment methods. BP patients are mostly the elderly, and they frequently have additional underlying diseases that limit their treatment options. Furthermore, some patients develop treatment resistance, making it difficult to control their condition even when using a variety of different treatment strategies. T helper 2 cell (Th2)‐related cytokines and chemokines, primarily interleukin‐4 (IL‐4), IL‐5, CCL‐13, CCL‐18, eotaxin, and others were found to be overexpressed in pemphigoid lesions.^1^ Dupilumab works therapeutically by inhibiting the downstream inflammatory response by blocking the IL‐4 receptor α (IL‐4R α) of IL‐4 and IL‐13.^2^ In this study, we retrospectively analyzed patients who were treated with dupilumab in our outpatient and inpatient departments between October 2020 and October 2022. The patients were mostly elderly and several of them had significant comorbidities. We examine the use, efficacy, and safety of dupilumab in this paper.

## 2 PATIENTS AND METHODS

### 2.1 Research patients

From October 2020 to October 2022, BP patients were treated with dupilumab in the dermatology department of the Second Affiliated Hospital of Kunming Medical University. *Inclusion criteria*: ① BP patients who were diagnosed with clinical findings, histopathology, immunofluorescence and/or enzyme‐linked immunosorbent assay showing positive anti‐BP180 and/or BP230 antibodies; ② Complete clinical medical records. *Exclusion criteria*: Patients who did not complete a full 12 weeks of regular follow‐up after dupilumab treatment.

### 2.2 Treatment and follow‐up

For the treatment with dupilumab, the atopic dermatitis treatment plan in the drug manual was consulted. The initial dose was 600 mg subcutaneously injected, followed by 300 mg injected every 2 weeks. Some patients with severe conditions were given medication weekly at first, then every two weeks after their disease was under control. Past use of hormones, immunosuppressants, anti‐inflammatory drugs (minocycline), and topical drugs by patients remained unchanged, and the dosage was adjusted based on changes in the disease. Following the initial administration, all patients were followed‐up once every 1–2 weeks, and then once every 2–4 weeks after 1 month. Details about skin lesions, pruritus degree, bullous pemphigoid disease area index (BPDAI) score, peripheral blood eosinophil count, total serum IgE concentration, liver and kidney function, blood coagulation function, other test indexes, and possible adverse drug reactions were collected at each follow‐up.

### 2.3 Evaluation of the therapeutic effect

The disease process is defined as follows: (1) Treatment baseline: The time when doctors began BP treatment with dupilumab; (2) Disease control stage: There are no new active lesions, and the existing lesions have begun to heal or recover; (3) Consolidation treatment stage: No new lesions appeared within at least 2 weeks, approximately 80% of the lesions had healed, and the pruritus symptoms were mild. (4) Complete remission following treatment: The patient had no new rash or pruritus in the original rash at least 2 months after discontinuing the drug treatment.^3^

Definition of therapeutic effect: (1) Fully controlled: The area of active skin lesions decreased by more than 90% when compared to the baseline; (2) Mostly controlled: The area of active skin lesions decreased by 50%–90% when compared to the baseline; (3) Partially controlled: The area of active skin lesions decreased by 50% when compared to the baseline; (4) Uncontrolled: The original skin lesions did not change or were aggravated. (5) Treatment failure: The discontinuation of dupilumab treatment was due to disease progression, adverse reactions, or death.^4^, ^5^ The short‐term and medium‐term efficacy rates of dupilumab were assessed 2 and 12 weeks after the initial injection, respectively.

### 2.4 Statistical method

SPSS 26.0 statistical software was used to process the data in this study. The measurement data were expressed by M (Q1, Q3) when it did not accord with the normal distribution, and by ($\bar{x}$ ± s) when it was consistent with the normal distribution. The paired t‐test was used to compare measurement data that fit the normal distribution, while Wilcoxon and Friedman's tests were used to compare measurement data that did not fit the normal distribution. The difference was statistically significant at *p* < .05.

## 3 RESULTS

### 3.1 Clinical data baseline characteristics

Data on 11 BP patients (4 males and 7 females) treated with dupilumab were collected, with a median age of 79 (45, 92) years, a median disease duration of 2 (1, 180) months, a BPDAI score of 113 (62, 181), and a pemphigoid pruritus visual analog scale score of (24.9091 ± 4.98908) (Table 1).

**Table 1:** Therapeutic drug use of patients at baseline.

Patient code	Sex	Age	Course of disease (month)	Glucocorticoid use at baseline (as calculated by prednisone)	Minocycline hydrochloride	Niacinamide	Cyclosporine	Mycophenolate mofetil	Albumin	Immunoglobulin	Topical drug (cream halometasone/triclosan)
Case1	Female	76	3	0							√
Case2	Female	73	12	30 mg	√				√		√
Case3	Male	91	180	37.5 mg	√	√					√
Case4	Female	79	1	37.5 mg		√	√				√
Case5	Female	45	6	150 mg	√	√				√	√
Case6	Male	80		30 mg							√
Case7	Female	84	1	30 mg				√			√
Case8	Female	90	4	50 mg							√
Case9	Female	67	5	50 mg							√
Case10	Male	81	3	50 mg	√				√	√	√
Case11	Male	69	2	0	√						√

During the course of the dupilumab treatment, one patient was not treated with hormones, while the other patients received hormone treatment. The minimum dose (prednisone equivalent) was 20 mg and the maximum dose was 100 mg. Except for one patient, all others received topical hormone therapy (halometasone ointment or halometasone/triclosan ointment). Minocycline hydrochloride capsule was used concurrently with the aforementioned topical hormone in nine patients; intravenous human immunoglobulin 25 g/day (for 2 days) was used concurrently in one patient, and cyclosporine, a soft capsule was used concurrently in one patient. The majority of patients had one or more comorbidities, with only one patient having no comorbidity. Comorbidities recorded included hypertension, myocardial infarction, pulmonary infection, old pulmonary tuberculosis, cerebral hemorrhage, cerebral infarction, brain atrophy, lower limb atherosclerosis, pleural effusion, pericardial effusion, mediastinal lymph node enlargement, pleural adhesion, pleural calcification, hepatic cyst, renal cyst, atrial tachycardia, atrial premature beat, complete right bundle branch block, abnormal electrocardiogram, hypoproteinemia, hypocalcemia, hyponatremia, digestive tract ulcer, diabetes, cataract, and others.

### 3.2 Disease control

#### 3.2.1 BPDAI score

**Figure 1:**
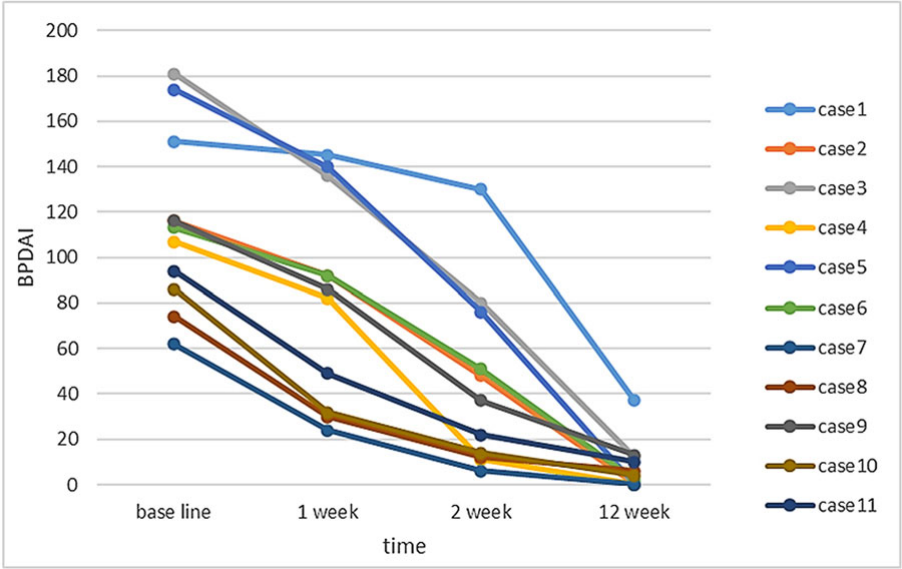
Changes in the BPDAI score after dupilumab treatment: With the passage of time, the BPDAI score gradually decreased. The Friedman test revealed that the BPDAI score decreased significantly 1 week after treatment (*Z* = −2.936, *p* = .003), and at 12 weeks, the majority of patients were fully or mostly controlled (*Z* = −2.937, *p* = .03). BPDAI, bullous pemphigoid disease area index.

The patients quickly entered the disease control stage after receiving dupilumab treatment for BP. After 2 weeks of treatment with dupilumab, the rash in 10 (90.91%) of the 11 cases was fully or mostly controlled. One case had poor rash control after 3 weeks of dupilumab treatment, but the rash was controlled when prednisone tablets (30 mg/day) were used concurrently. The rash in one patient completely disappeared in the eighth week of follow‐up but the patient refused to continue treatment and was treated with prednisone 15 mg alone; there was no recurrence of the rash after 24 weeks of follow‐up and the treatment was continued with hormone 7.5 mg. No new lesions appeared in the patients during the consolidation treatment stage. At present, all drug treatments have been discontinued for at least 2 months, and there has been no recurrence. Following treatment, three patients achieved complete remission. The BPDAI score dropped from 113 (62, 181) at baseline to 37 (6, 130) at 2 weeks (*Z* = −3.059, *p* = .002), and decreased to 4 (0, 37) at 12 weeks after treatment (*Z* = −2.937, *p* = .003, *p* = .003; Figure 1).

#### 3.2.2 Pemphigoid pruritus visual analog scale (VAS) score

**Figure 2:**
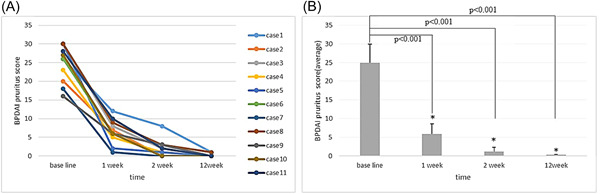
Changes in the pruritus score on the visual analog scale in pemphigoid patients: (A) changes in pruritus score on the visual analog scale of patients at baseline, 1 week after treatment and 2 weeks after treatment; (B) after treatment, the skin pruritus was significantly different from baseline at 1 week (*t* = 13.177, *p* < .001) and 2 weeks (*t* = 15.925, *p* < .001).

At baseline, all patients had varying degrees of pruritus with the most severe case scoring a full 30 points. After treatment with dupilumab, the pruritus sensation of the skin rash was quickly relieved. The 11 patients experienced relief from pruritus after receiving dupilumab for 0–3 days (0.5, 7) days. Pruritus began to subside to varying degrees in 8 patients after 1 day of treatment with dupilumab. The pruritus in all patients improved significantly after 2 weeks of treatment (*t* = 15.925, *p* < .001, Figure 2).

#### 3.2.3 Dermatology life quality index (DLQI) score

**Figure 3:**
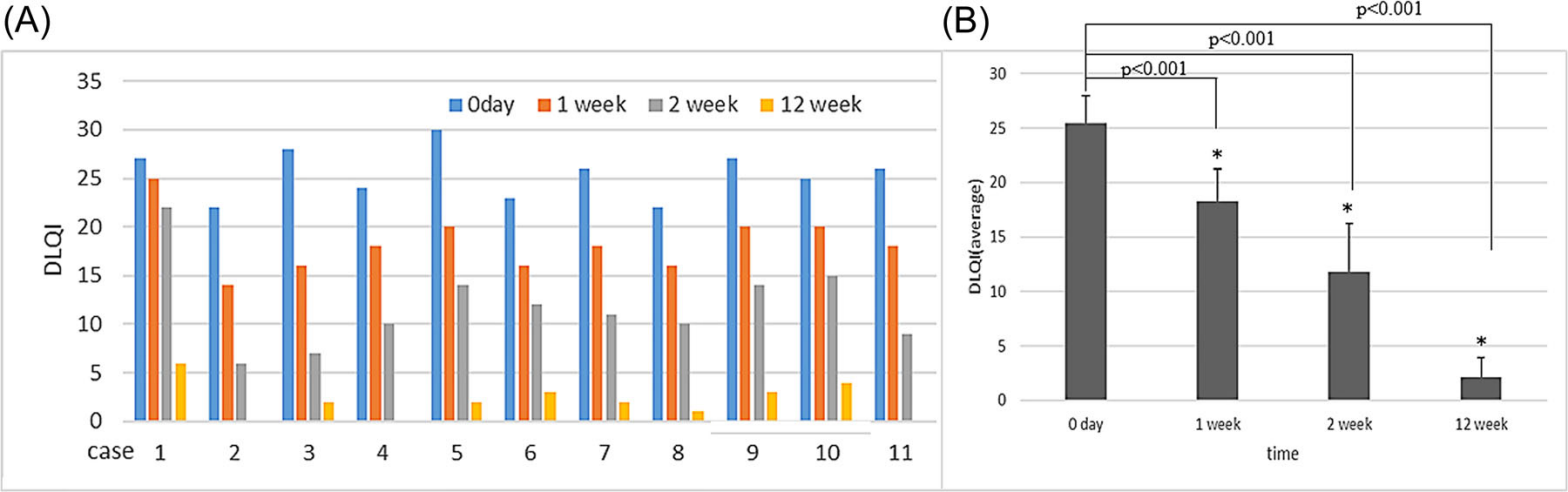
Changes in the dermatology life quality index (DLQI) of the 11 patients: (A) The dermatology life quality of 11 pemphigoid patients was severely impacted. (B) The DLQI score of the patients decreased from (25.5 ± 2.5) at the time of the diagnosis to (11.8 ± 4.4) at 2 weeks (*t* = 10.764, *p* < .001), and to (2.1 ± 1.9) at 12 weeks (*t* = 30.038, *p* < .001).

The dermatology life quality of pemphigoid patients was significantly improved. The DLQI score of pemphigoid patients decreased from (25.5 ± 2.5) at the time of diagnosis to (11.8 ± 4.4) at 2 weeks (*t* = 10.764, *p* < .001), and to (2.1 ± 1.9) at 12 weeks (*t* = 30.038, *p* < .001) (Figure 3).

### 3.3 Changes in laboratory indicators

#### 3.3.1 Changes in the eosinophil count and total IgE

The changes in the peripheral blood eosinophil count and total IgE were recorded for 10 patients. Among them, two patients had elevated peripheral blood eosinophils and two patients had a higher baseline total IgE concentration than the normal value. In these 4 patients, peripheral blood eosinophil count and total IgE levels decreased after treatment with dupilumab.

#### 3.3.2 Changes in blood coagulation function

Nine of the patients had abnormal indexes of blood coagulation function, and eight of the nine patients had simultaneous elevations of prothrombin activity, fibrinogen degradation products (FDPs), and thrombus dissolved dimer (DD). In one of the nine patients, the prothrombin activity was slightly elevated. The corresponding indicators tended to be normal after treatment and as the disease improved.

#### 3.3.3 Changes in total blood protein

Total blood protein decreased in 10 patients to varying degrees, while albumin decreased in three cases.

### 3.4 Adverse reactions

No adverse reactions such as conjunctivitis, blepharitis, eye pruritus, allergic reaction at the injection site, headache, eosinophilia, and oral herpes were detected in the 11 patients.

### 3.5 Follow‐up treatment plan

Two patients stopped taking dupilumab after a full 40‐week treatment (involving 21 drugs). After 60 weeks of follow‐up, these 2 patients had no recurrences and stopped taking all the medications. One patient's rash was resolved completely by the eighth week of follow‐up. The patient refused maintenance therapy and was only given prednisone 15 mg. There was no recurrence of the rash after 24 weeks of follow up, and this patient experienced complete relief from the ailment and stopped taking medication. One patient was treated with dupilumab alone for 4 weeks. Although there was no new rash, the old rash did not subside. The rash began to improve after prednisone tablets of 30 mg/day were given. Before starting dupilumab, one patient received a methylprednisolone injection of 80 mg/day, and a large number of new rashes continued to develop. There was some relief from the trunk rash after treatment with dupilumab, but there were still a large number of new blisters on limbs, hands, and feet. Following that, the hormone dosage was increased to 120 mg/day of methylprednisolone injection and to 300 mg/week of dupilumab. The rash was then controlled, and the pruritus was relieved.

## 4 DISCUSSION

The production of autoantibodies against hemidesmosome components is central to the pathogenesis of BP. Antibody production and function are closely linked to the inflammatory response mediated by Th2. Activated Th2 cells can secrete cytokines such as IL⁃4, IL⁃5, IL⁃13, IL‐31, and chemokines. On the one hand, they can recruit eosinophils and other inflammatory cells and promote B cells to convert and secrete IgE. The release of inflammatory factors, on the other hand, maintains the continuous activation of the inflammatory response via positive feedback. Furthermore, cytokines like IL‐4 and IL‐31 can sensitize peripheral nerves causing them to mediate pruritus sensation.^6^ Dupilumab has the ability to bind to the IL⁃4 R α receptor, inhibit the action of IL‐4 and IL⁃13, and inhibit downstream signal transduction, thereby inhibiting inflammatory response, reducing pruritus, and preventing disease progression.^7^ Dupilumab is not currently approved for the treatment of BP. However, due to the role of Th2‐mediated inflammation in the pathogenesis of BP, research initiatives around the world^8^, ^9^, ^10^, ^11^, ^12^, ^13^, ^14^, ^15^, ^16^, ^17^, ^18^, ^19^, ^20^, ^21^, ^22^, ^23^ and within China^24^, ^25^, ^26^, ^27^ have used dupilumab to treat refractory or moderate to severe BP.

We attempted to use dupilumab to treat 11 BP patients whose underlying diseases made conventional treatment ineffective or who had poor responses to conventional treatment, and we achieved positive results. In terms of dupilumab use in BP, we currently referred to its use in the treatment of atopic dermatitis. Ten of the 11 patients were treated with hormones concurrently. However, the reduction in hormone dosage after disease control was faster than that for the patient who was conventionally treated with hormones alone, and there was no disease rebound. As a result, the use of dupilumab is of immense significance in reducing hormone dosage and rapidly reducing hormone frequency. The condition of a 43‐year‐old patient was out of control following an active radical treatment protocol. We achieved good results by shortening the dupilumab use cycle and switching to weekly dosing when adding dupilumab. It is worth noting that a 91‐year‐old patient had more than 80% skin involvement. Erythema, blisters, blood blisters, and bullae were widely distributed all over the skin. The rash could not be controlled with dupilumab and topical halometasone cream for 4 weeks. The rash was controlled with 30 mg/day prednisone tablets. This suggests that combining hormones and dupilumab could be an effective way to control the disease in patients with severe conditions and obvious inflammatory responses to rash. However, due to the small number of cases, the sample size must be increased for further observations and analysis.

The first benefit that patients noticed after receiving dupilumab treatment was the improvement in pruritus. This medication controlled BP pruritus symptoms faster than it improved skin lesions. Except for one patient, pruritus in the other patients were relieved to varying degrees on the day of medication and was completely relieved within 2 weeks. The relief from pruritus improved the sleep quality of patients and effectively improved their life quality.

In recent years, there has been increasing evidence on the important role eosinophils (EOS) and immunoglobulin E (IgE) play in the pathogenesis of BP. EOS is a cell essential for blister formation mediated by IgE autoantibodies.^28^ It was reported that 50% of BP patients had elevated EOS in their peripheral blood.^29^ In untreated BP patients, the number of EOS cells was linearly correlated with disease severity.^30^ Only two patients in this study had eosinophilia, which may be attributable to the small sample size. On the other hand, it could be due to the fact that most of these patients had used glucocorticoids before admission. The peripheral blood eosinophil count and total IgE were elevated, which may be related to the small sample size. Following treatment, the blood eosinophil count and total IgE concentration of the two patients returned to normal, which was consistent with the other cases.

Furthermore, the enrolled patients had varying degrees of blood coagulation dysfunction, primarily an increase in D dimer and fibrinogen degradation products (FDPs). The skin is closely related to the hemorrhagic and blood coagulation systems. Skin epidermal cells can secrete tissue factors that induce blood coagulation. At the same time, the fibrinolytic system can be activated to help with anticoagulation. Blood coagulation and the fibrinolytic system activation in the peripheral blood of BP patients leads to increased vascular permeability, making it easier for inflammatory cells to migrate from blood vessels to skin tissues. Infiltration of skin inflammatory cells can aggravate local inflammatory response, promoting blister formation,^31^ but it also increases the risk of thrombosis.^32^ D dimer and FDPs are specific fibrinolysis markers. D‐dimer and FDPs increased during disease activity and decreased to normal after disease remission in the patients studied, indicating that blood coagulation and fibrinolytic function were activated and abnormal while the disease was active. The specific mechanism remains unclear.

Total blood protein decreased to varying degrees in 10 of the patients, while albumin decreased concurrently in 3 patients. With disease remission, blood protein levels gradually returned to normal. A large area of skin rash, erosion, ulceration, protein loss, malnutrition, and increased consumption were the main considerations for the above changes. Following disease relief, the condition gradually returned to normal.

## 5 CONCLUSION

To summarize, dupilumab therapy can achieve disease control in BP, effectively relieve pruritus symptoms, and is relatively safe. The excellent safety profile of dupilumab, which does not suppress immunity, unlike other therapeutic options. It can effectively reduce the dosage of hormones and shorten the use cycle for patients on hormone medication, providing a new treatment option for patients with poor control or intolerance to conventional treatment. However, given the short history of dupilumab in the treatment of BP, the long‐term efficacy of the drug must be monitored. The following limitations apply to this study: small sample size, no control group, and short follow‐up time. Future research should increase the sample size, implement reasonable controls, and investigate the specific use of dupilumab.

## AUTHOR CONTRIBUTIONS

**Lingyu Hu**: Data curation; resources; writing—original draft; writing—review & editing. **Ruiting Huang**: Data curation; methodology; resources; writing—review & editing. **Fuqiong Jiang**: Formal analysis; funding acquisition; methodology; resources; writing—original draft; writing—review & editing. **Shuqiong You**: Data curation; formal analysis; resources; writing—original draft. **Qian Wu**: Data curation; methodology; resources; writing—review & editing.

## CONFLICT OF INTEREST STATEMENT

The authors declare no conflict of interest.

## ETHICS STATEMENT

This study was conducted with approval from the Ethics Committee of Second Affiliated Hospital of Kunming Medical University. This study was conducted in accordance with the declaration of Helsinki. Written informed consent was obtained from all participants.

## Data Availability

The datasets used and/or analyzed during the current study are available from the corresponding author on reasonable request.

## References

[1] Giomi B , Caproni M , Calzolari A , Bianchi B , Fabbri P . Th1, Th2 and Th3 cytokines in the pathogenesis of bullous pemphigoid. J Dermatol Sci. 2002;30(2):116‐128. 10.1016/s0923-1811(02)00067-1 12413767

[2] Simpson EL , Bieber T , Guttman‐Yassky E , et al. Two phase 3 trials of dupilumab versus placebo in atopic dermatitis. N Engl J Med. 2016;375(24):2335‐2348. 10.1056/NEJMoa1610020 27690741

[3] Viti G , Forcella C , Feliciani C , Murrell DF . Beyond the skin: disease parameters in pemphigus. Italian J Dermatol Venereol. 2021;156(2):147‐150. 10.23736/S2784-8671.21.06678-5 33960750

[4] Egami S , Yamagami J , Amagai M . Autoimmune bullous skin diseases, pemphigus and pemphigoid. J Allergy Clin Immunol. 2020;145(4):1031‐1047. 10.1016/j.jaci.2020.02.013 32272980

[5] Montagnon CM , Lehman JS , Murrell DF , Camilleri MJ , Tolkachjov SN . Intraepithelial autoimmune bullous dermatoses disease activity assessment and therapy. J Am Acad Dermatol. 2021;84(6):1523‐1537. 10.1016/j.jaad.2021.02.073 33684497

[6] Hashimoto T , Kursewicz CD , Fayne RA , et al. Pathophysiologic mechanisms of itch in bullous pemphigoid. J Am Acad Dermatol. 2020;83(1):53‐62. 10.1016/j.jaad.2019.07.060 31351883

[7] Russo R , Cozzani E , Gasparini G , Parodi A . Targeting interleukin 4 receptor α: a new approach to the treatment of cutaneous autoimmune bullous diseases? Dermatol Ther. 2020;33(1):e13190. 10.1111/dth.13190 31863534 PMC7154653

[8] Riquelme‐Mc Loughlin C , Mascaró JM . Treatment of pemphigoid gestationis with dupilumab. Clin Exp Dermatol. 2021;46(8):1578‐1579. 10.1111/ced.14765 34048080

[9] Kaye A , Gordon SC , Deverapalli SC , Her MJ , Rosmarin D . Dupilumab for the treatment of recalcitrant bullous pemphigoid. JAMA Dermatol. 2018;154(10):1225‐1226. 10.1001/jamadermatol.2018.2526 30140849

[10] Zhang Y , Xu Q , Chen L , et al. Efficacy and safety of dupilumab in moderate‐to‐severe bullous pemphigoid. Front Immunol. 2021;12:738907. 10.3389/fimmu.2021.738907 34721404 PMC8552038

[11] Seyed Jafari SM , Feldmeyer L , Bossart S , Simon D , Schlapbach C , Borradori L . Case report: combination of omalizumab and dupilumab for recalcitrant bullous pemphigoid. Front Immunol. 2021;11:611549. 10.3389/fimmu.2020.611549 33584689 PMC7879677

[12] Cao P , Xu W , Zhang L . Rituximab, omalizumab, and dupilumab treatment outcomes in bullous pemphigoid: a systematic review. Front Immunol. 2022;13:928621. 10.3389/fimmu.2022.928621 35769474 PMC9235912

[13] Wang SH , Zuo YG . Commentary: efficacy and safety of dupilumab in moderate‐to‐severe bullous pemphigoid. Front Immunol. 2021;12:800609. 10.3389/fimmu.2021.800609 34887875 PMC8649839

[14] Velin M , Dugourd PM , Sanchez A , Bahadoran P , Montaudié H , Passeron T . Efficacy and safety of methotrexate, omalizumab and dupilumab for bullous pemphigoid in patients resistant or contraindicated to oral steroids. A monocentric real‐life study. J Eur Acad Dermatol Venereol. 2022;36(7):e539‐e542. 10.1111/jdv.17999 35143077

[15] Jendoubi F , Bost C , Tournier E , Paul C , Konstantinou MP . Severe pemphigoid nodularis successfully treated with dupilumab. Dermatol Ther. 2022;35(9):e15727. 10.1111/dth.15727 35861645 PMC9539641

[16] Wang M , Wang J , Shi B . Case report: dupilumab for the treatment of bullous pemphigoid. Dermatol Ther. 2022;35(7):e15541. 10.1111/dth.15541 35478478

[17] Russo R , Capurro N , Cozzani E , Parodi A . Use of dupilumab in bullous pemphigoid: where are we now? J Clin Med. 2022;11(12):3367. 10.3390/jcm11123367 35743438 PMC9224939

[18] Valenti M , De Giacomo P , Lavecchia A , Valenti G . A severe case of IgA bullous pemphigoid successfully treated with dupilumab. Dermatol Ther. 2022;35:e15890. 10.1111/dth.15890 36181411

[19] Shan Y , Zuo YG . A successful case of vesicular pemphigoid concurrent with pulmonary tuberculosis with dupilumab. Dermatol Ther. 2022;35(4):e15330. 10.1111/dth.15330 35080079

[20] Takamura S , Teraki Y . Treatment of bullous pemphigoid with dupilumab: dupilumab exerts its effect by primarily suppressing T‐helper 2 cytokines. J Dermatol. 2022;49(9):845‐850. 10.1111/1346-8138.16428 35538742

[21] Li W , Cai S , Man X . The treatment of refractory atypical bullous pemphigoid with generalized eczema and intense pruritus with dupilumab. Dermatol Ther. 2021;35(2):e15243. 10.1111/dth.15243 34854196

[22] Yang J , Gao H , Zhang Z , et al. Dupilumab combined with low‐dose systemic steroid therapy improves efficacy and safety for bullous pemphigoid. Dermatol Ther. 2022;35(8):e15648. 10.1111/dth.15648 35715972

[23] Pop SR , Strock D , Smith RJ . Dupilumab for the treatment of pembrolizumab‐induced bullous pemphigoid: a case report. Dermatol Ther. 2022;35(8):e15623. 10.1111/dth.15623 35669992 PMC9539473

[24] Ji J , Xu HX , Feng SY , Xu CC . Dupilumab for bullous pemphigoid: a case report and literature review. Zhong Guo Lin Chuang Yan Jiu. 2022;35(5):705‐707. 10.13429/j.cnki.cjcr.2022.05.023

[25] Pan CM , Pan M , Feng YM , Ding GZ , Zhu YP , Sun L . Dupilumab for refractory bullous pemphigoid: a case report. Zhong Guo Pi Fu Xing Bing Xue Za Zhi. 2022;36(3):311‐314. 10.13735/j.cjdv.1001-7089.202106005

[26] Yang L , Zeng YP , Pu HZ . Dupilumab combined with glucocorticoids in the treatment of bullous pemphigoid: first report and literature review in China. Chinese J Clin Immunity Allergy. 2021;15(1):47‐52. 10.3969/j.issn.1673-8705.2021.01.009

[27] Zhao LQ , Chen Y , Chen DY , et al. Efficacy and safety of dupilumab in the treatment of 21 cases of bullous pemphigoid: a retrospective study. Chinese J Dermatol. 2022;55(6):480‐485. 10.35541/cjd.20210813

[28] Lin L , Hwang BJ , Culton DA , et al. Eosinophils mediate tissue injury in the autoimmune skin disease bullous pemphigoid. J Invest Dermatol. 2018;138(5):1032‐1043. 10.1016/j.jid.2017.11.031 29246800 PMC7531612

[29] Kridin K . Peripheral eosinophilia in bullous pemphigoid: prevalence and influence on the clinical manifestation. Br J Dermatol. 2018;179(5):1141‐1147. 10.1111/bjd.16679 29663327

[30] van Beek N , Schulze FS , Zillikens D , Schmidt E . IgE‐mediated mechanisms in bullous pemphigoid and other autoimmune bullous diseases. Expert Rev Clin Immunol. 2015;12(3):267‐277. 10.1586/1744666X.2016.1123092 26588556

[31] Marzano AV , Tedeschi A , Berti E , Fanoni D , Crosti C , Cugno M . Activation of coagulation in bullous pemphigoid and other eosinophil‐related inflammatory skin diseases. Clin Exp Immunol. 2011;165(1):44‐50. 10.1111/j.1365-2249.2011.04391.x 21488867 PMC3110320

[32] Cugno M , Marzano AV , Bucciarelli P , et al. Increased risk of venous thromboembolism in patients with bullous pemphigoid. the inventep (incidence of venous thromboembolism in bullous pemphigoid) study. Thromb Haemost. 2016;115(1):193‐199. 10.1160/TH15-04-0309 26245987

